# Supplementary material for: Analyzing preventive precautions to limit spread of COVID-19
Source: PLoS One. 2022 Aug 24;17(8):e0272350. doi: 10.1371/journal.pone.0272350 (PMC9401132; doi:10.1371/journal.pone.0272350)
Supplement: Attachment — Submitted filename: Response to reviewers comments.pdf [file pone.0272350.s001.pdf]

# Response to Comments

**Manuscript ID**: PONE-D-22-09735

**Title**: Analyzing Preventive Precautions to Limit Spread of COVID-19

**Authors**: Ayaz Ahmad, Furqan Rustam, Eysha Saad, Muhammad Abubakar Siddique, Ernesto Lee, Arturo Ortega Mansilla, Isabel de la Torre Díez and Imran Ashraf*

Dear Editor,

Thank you very much for allowing us to revise the manuscript. We would like to thank the editor and all the reviewers for their valuable comments and suggestions. Based on the feedback, we have revised our manuscript. The detailed modifications to address reviewers' comments are provided in the following. For clarity, we have marked our responses in blue. Whenever we copy a paragraph from the manuscript here, we mark it as a red color.

## Answers to Comments

### REVIEWER 1

**Concern 1:** The Introduction should highlight the relevance of the topic, the novelty of the results, the importance of policy implications, the sample's choice, the methodology's appropriateness, the data used, the contribution to the literature, and the limitations of the study.

**Response**: We would like to thank the reviewer for the valuable comments to improve the quality of the manuscript. We make changes in the manuscript according to reviewer concerns. In the light of the reviewer's comments, the following changes have been made.

**Page 2 of the revised manuscript**

Keeping in view the surge in the infected people, health institutions and governments introduced corresponding safety precautions to curb the spread of the virus such as keeping a distance of 6 feet,

sanitizing the area and persons, wearing facemasks, etc. [11]. Such precautionary measures are imposed by the Governments to limit the spread of COVID-19 as they are the first defense against the pandemic.

**Page 2 of the revised manuscript**

Previous studies manifested that the spread of the virus can be minimized if the public follows the precautionary measures defined by the government and healthcare officials [14-16]. Although COVID-19 policies are designed and deployed for the safety and well-being of people; such policies often create disputes and dissent. A large number of people demonstrate an unwillingness to follow such precautions, especially wearing the facemask [17, 18]. People think that wearing a face mask is difficult and may lead to additional respiratory complications if used for a longer period. So, investigating the views of the masses regarding such restrictions is an important research area to revise policies to both resolves people's disagreements and protect them from the disease.

**Page 3 of the revised manuscript**

Predominantly, existing studies follow machine learning-based approaches for analyzing the sentiments for several reasons. Datasets contain a large number of tweets, views, and short texts, and manual annotation and analysis of such a large dataset are laborious and time-consuming. Such issues can be resolved by using machine learning models. Human experts are subject to error, and bias due to mood, fatigue, and other factors which may lead to wrong annotation and sentiment classification. Also, the performance of machine learning can be enhanced by following parameter optimization, data cleaning, and appropriate feature selection. Given these advantages, and embracing the use of machine learning approaches by existing studies, this research adopts a machine-learning-based methodology. It follows the similar flow of data preprocessing, feature extraction, training, and evaluation of machine learning models as embraced by existing studies. This methodology eventually leads to two advantages in essence. First, the study follows the norms of the research community that has been well established. Secondly, the use of the same benchmark makes it easy to analyze its performance with respect to existing works.

The objectives and contributions of the study have also been revised.

**Page 3 of the revised manuscript**

This study proposes a hybrid approach to perform sentiment analysis of tweets related to safety precautions for COVID-19 and makes the following contributions

- A unified framework of sentiment lexicon and machine learning classifier has been proposed where the proposed model is a voting classifier, extreme boosting-decision tree (EB-DT), and integrates extra tree classifier (ETC), gradient boosting machine (GBM), and DT models. For experiments, different sets of data are extracted from Twitter. Three different datasets are extracted, to be precise, for analyzing the change in peoples' sentiments over time.
- Machine learning models are also employed for performance comparison including AdaBoost classifier (AC), ETC, logistic regression (LR), random forest (RF), GBM, K nearest neighbors (KNN), support vector classifier (SVC), and DT which are used with term frequency-inverse document frequency (TF-IDF) and bag of words (BoW). Experiments also involve long short-term memory (LSTM), convolutional neural network (CNN)-LSTM, and gated recurrent unit (GRU) with a large dataset collected from Twitter. The lexicon-based TextBlob is used to annotate the collected dataset.

- Topic modeling of positive and negative is carried out using the Latent Dirichlet allocation (LDA) algorithm to analyze the factors that negatively influence the safety precautions against COVID-19.
- The accuracy of the proposed framework regarding the sentiment classification is compared to a professional sentiment analysis app called 'sentiment viz app'.

**Concern 2:** The literature review is partial and incomplete, and some recent and relevant contributions should be cited and discussed: i.e., 10.1017/S095026882100248X; 10.1016/j.jenvman.2021.112241; 10.1007/s11356-020-10689-0; 10.1016/j.envres.2020.110663; 10.1016/j.apenergy.2020.115835.

**Response**: Authors are grateful for your valuable suggestions. The suggested articles are discussed in the 'Related Work' Section of the revised manuscript, as follows.

**Page 5 of the revised manuscript**

On top of sentiment analysis works, several studies investigate the relationships between various environmental factors and COVID-19. For example, [35] proposed an approach to find the relationship between COVID-19-related deaths, PM10, economic growth, PM2.5, and NO2 concentrations in New York State. The authors used city-level data to analyze the pattern using machine learning models. Similarly, [36] adopted a machine learning approach to find the relationship between pollution, economic growth, and deaths in India. The influence of NO2 concentration on COVID-19 deaths is investigated in [37] within the context of France. An artificial neural network has been adopted with a causal direction from the dependency algorithm for the study. The study successfully determines the NO2 thresholds concerning the spread of COVID-19. Similarly, the influence of particulate matter on public health is investigated in [38] for French cities.

**References**

[35] Magazzino, C., Mele, M. and Sarkodie, S.A., 2021. The nexus between COVID-19 deaths, air pollution and economic growth in New York state: Evidence from Deep Machine Learning. *Journal of Environmental Management*, *286*, p.112241.
[36] Mele, M. and Magazzino, C., 2021. Pollution, economic growth, and COVID-19 deaths in India: a machine learning evidence. *Environmental Science and Pollution Research*, *28*(3), pp.2669-2677.
[37] Mele M, Magazzino C, Schneider N, Strezov V. $NO_2$ levels as a contributing factor to COVID-19 deaths: The first empirical estimate of threshold values. Environ Res. 2021 Mar;194:110663. doi: 10.1016/j.envres.2020.110663. Epub 2021 Jan 5. PMID: 33417906; PMCID: PMC7783466.
[38] Magazzino C, Mele M, Schneider N. The relationship between air pollution and COVID-19-related deaths: An application to three French cities. Appl Energy. 2020 Dec 1;279:115835. doi: 10.1016/j.apenergy.2020.115835. Epub 2020 Sep 12. PMID: 32952266; PMCID: PMC7486865.

**Concern 3**: The theoretical framework should be discussed more in detail.
**Response:** Thank you very much for your positive feedback and insightful comments. The proposed framework has been elaborated in the revised manuscript. Following is the newly added detail which is copied here for your convenience.

**Page 10~11 of the revised manuscript**

Each model in the proposed EB-DT ensemble takes the text input features to predict probabilities for positive, negative, and neutral sentiments. Mathematically it can be written as

$$C_p = (\frac{\sum_{i=1}^{N} avgC_i}{N}) \quad (1)$$

where $C_p$ is the prediction probability by the EB-DT using soft voting criteria, $N$ is the number of models, and $avgC$ is the per sentiment probability by each model.

The $C_p$ can also be written as

$$C_p = (\frac{C_{DT}+C_{GBM}+C_{ETC}}{N}) \quad (2)$$

$$Pos_p = (\frac{Pos_{DT}+Pos_{GBM}+Pos_{ETC}}{3}) \quad (3)$$

For EB-DT, Equation 1 is used to find the probability of positive sentiment against a tweet. Here $Pos_{DT}$ is the probability of positive class by the DT model, $Pos_{GBM}$ is the probability by the GBM, and $Pos_{ETC}$ is the probability of positive class by the ETC model. Similarly, EB-DT calculates the probability for negative and neutral sentiments as follows.

$$Neg_p = (\frac{Neg_{DT}+Neg_{GBM}+Neg_{ETC}}{3}) \quad (4)$$

$$Neu_p = (\frac{Neu_{DT}+Neu_{GBM}+Neu_{ETC}}{3}) \quad (5)$$

The ensemble model EB-DT makes the final prediction using

$$EB - DT_p = argmax\{Pos_p\,, Neg_p\,, Neu_p\} \quad (6)$$

The functioning of the EB-DT can be further elaborated using an example run. For this purpose, the tweet "One of two shots so far, but have always, and will continue to wear the mask" is taken. DT probabilities against this tweet are (Pos=0.3, Neg=0.2, Neu=0.5), ETC shows probabilities as (Pos=0.2, Neg=0.2, Neu=0.6), while GBM shows (Pos=0.2, Neg=0.1, Neu=0.7) for the given tweet. Using Equation 2, average probabilities can be calculated as

$$Pos_p = \left(\frac{0.3+0.2+0.2}{3}\right) = 0.2 \quad (7)$$

$$Neg_p = \left(\frac{0.2+0.2+0.1}{3}\right) = 0.16 \quad (8)$$

$$Neg_p = \left(\frac{0.5+0.6+0.7}{3}\right) = 0.6 \quad (9)$$

The average probabilities are then used by the argmax function to predict the final class of the tweet which in this case is neutral as it has the highest average probability considering the models that form the ensemble.

$$EB - DT_p = argmax\{0.23, 0.16, 0.6\} \quad (10)$$
$$EB - DT_p = 0.6\ (Neutral) \quad (11)$$

**Concern 4:** The estimated model must be justified in light of the literature on this specific topic.

**Concerns:** Thank you very much for your comments. The choice of the estimated model has been justified with respect to the existing literature. The newly added material is copied here for the convenience of the worthy reviewer.

**Page 10 of the revised manuscript**

The choice of an estimated ensemble model stands on several grounds. First, ensemble models have the tendency to show better performance as reported in the existing literature [32, 45, 55]. Utilizing more than one model potentially reduces the misclassification rate. Secondly, the choice of models varies concerning the nature of data and models can be influenced by the size of the feature set, the ratio of class samples (balanced vs imbalanced datasets), etc. which can increase the probability of model overfit or underfit. Using multiple models on the datasets and combining their output mitigates such risks. Thirdly, the voting criterion is selected because of the results reported for voting classifiers where the ensemble models tend to show better performance than individual models [33, 56]. Keeping in view the diversity of the text tokens used in the tweets, it seems a probable choice to utilize multiple classifiers. Different types of machine learning models like tree-based or linear models, show a different level of performance with sentiment analysis tasks, and using more than one classifier to make the final prediction tends to increase the probability of correct class prediction.

**References**

[32] Ozcift A. Medical Sentiment Analysis Based on Soft Voting Ensemble Algorithm. Yonetim Bilisim Sistemleri Dergisi. 2020, 6(1):42-50.
[33] Khalid et al., GBSVM: sentiment classification from unstructured reviews using ensemble classifier. Applied Sciences. 2020, 10(8):2788.
[45] Rupapara et al., Impact of SMOTE on imbalanced text features for toxic comments classification using RVVC model. IEEE Access. 2021, 9:78621-78634.
[55] Ganaie M, Hu M, et al. Ensemble deep learning: A review. arXiv preprint arXiv:210402395. 2021.
[56 Mehmood A, On BW, Lee I, Ashraf I, Choi GS. Spam comments prediction using stacking with ensemble learning. In: Journal of Physics: Conference Series. vol. 933. IOP Publishing; 2017, 012012.

**Concern 5**: Descriptive statistics are absent.

**Response:** Statistics are provided in Figure 2 and Figure 3 of the revised manuscript. A brief description is also provided on Page 7~8 of the revised manuscript.

**Page 7~8 of the revised manuscript**

We categorize the data into three classes positive, negative, and neutral. Among a total of 8911 records, TextBlob categorized 3193 records as positive, 4599 records as neutral while 1119 are labeled as negative, as shown in Figure 2.

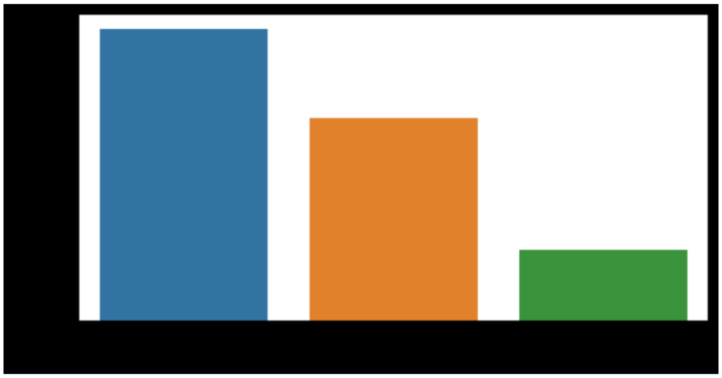

**Figure 2:** Number of samples for neutral, positive, and negative sentiments by TextBlob.

Figure 3 shows the ratios of positive, negative, and neutral sentiments, as labeled by TextBlob. It can be observed that neutral sentiments make up most of the dataset with 51.61% of the dataset. It is followed by the positive and negative tweets which make up 35.83% and 12.56%, respectively.

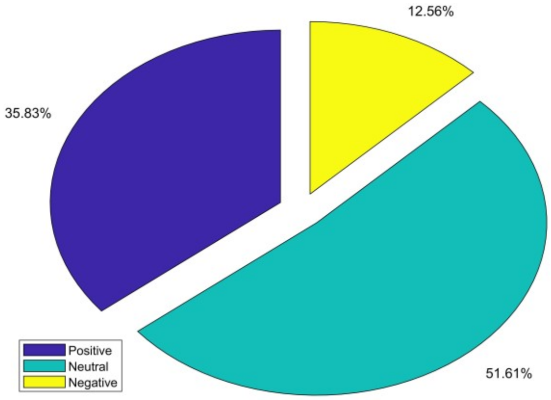


**Figure 3:** Ratio of positive, negative, and neutral sentiments.

**Concern 6**: Diagnostic tests are absent.
**Response:** We appreciate the reviewer's concern, however, the current study aims at analyzing the sentiments of the people using the Tweets posted on Twitter. No diagnostic tests are performed for the study.

**Concern 7**: Robustness checks are absent.
**Response:** Thank you very much for your comments. For analyzing the robustness of the proposed approach, we compare its performance regarding computational complexity which is measured in terms of time required for prediction in comparison to existing works. These results are presented in Table 8 of the revised manuscript.

**Page 17 of the revised manuscript**

The proposed model is better in terms of computational time as compared to other studies. Although [34] provides a much smaller execution time, its accuracy is substantially low as compared to the proposed model. Also, the current study uses three models while existing studies use two models for the ensemble.

**Table 8.** Performance comparison with previous studies.

| Reference | Model | Accuracy | Precision | Recall | F1 score | Execution time |
|---|---|---|---|---|---|---|
| [32] | SVE | 0.97 | 0.97 | 0.95 | 0.96 | 17.6 Sec |
| [33] | GBSVM | 0.96 | 0.95 | 0.95 | 0.95 | 16.2 Sec |
| [34] | LR-SGDC | 0.93 | 0.95 | 0.91 | 0.92 | 6 Sec |
| Current study | EB-DT | 0.99 | 0.99 | 0.99 | 0.99 | 16.1 Sec |

**References**


[32] Ozcift A. Medical Sentiment Analysis Based on Soft Voting Ensemble Algorithm. Yonetim Bilisim Sistemleri Dergisi. 2020, 6(1):42-50.

[33] Khalid et al., GBSVM: sentiment classification from unstructured reviews using ensemble classifier. Applied Sciences. 2020, 10(8):2788.

[34] Rustam et al., Tweets classification on the base of sentiments for US airline companies. Entropy. 2019;21(11):1078.


**Concern 8:** The results should be discussed more in detail.

**Response:** We would like to thank the reviewer for the valuable comments to improve the quality of the manuscript. We added more details about the results according to the reviewer's suggestions.

**Concern 9**: Comparisons with previous studies are absent.

**Response:** Authors highly appreciate your suggestions. The manuscript is revised by adding a performance comparison Section. The newly added part is copied here for your convenience.

**Proposed Approach Comparison With Previous Studies**

To show the significance of the proposed approach, the performance comparison is carried out with existing state-of-the-art studies. Several studies are selected for comparisons, such as [32] proposed an ensemble model SVE for the medical review sentiment predictions. The study [33] used voting criteria to combine the GBM and SVM for user reviewer sentiment classification. Similarly, the study [34] used soft voting criteria to combine LR and SGDC models. Performance comparison results are presented in Table 8 indicating the superior performance of the proposed approach with a 0.99 accuracy score which is far better than existing works. In addition, the robustness of the proposed approach is evaluated by comparing its execution time.


**Table 8.** Performance comparison with previous studies.

| Reference | Model | Accuracy | Precision | Recall | F1 score | Execution time |
|---|---|---|---|---|---|---|
| [32] | SVE | 0.97 | 0.97 | 0.95 | 0.96 | 17.6 Sec |
| [33] | GBSVM | 0.96 | 0.95 | 0.95 | 0.95 | 16.2 Sec |
| [34] | LR-SGDC | 0.93 | 0.95 | 0.91 | 0.92 | 6 Sec |
| Current study | EB-DT | 0.99 | 0.99 | 0.99 | 0.99 | 16.1 Sec |

**Concern 10**: Conclusions are too short.
**Response:** The conclusion part is revised as suggested by the worthy reviewer.

**Page 19~20 of the revised manuscript**

The rapid COVID-19 outbreak inflicted large human and financial loss with the increasing number of infections that further continues to grow large with each passing day. The policy shifts by the government have resulted in the polarization of the underlying topic of the COVID-19 precautionary measures. The limited size of data in the case of surveys confines the scope of the study and a large dataset is required to analyze the trends over time. For this, this study gathered multiple datasets over different periods from Twitter containing tweets related to COVID-19 safety precautions. For analysis, an automated hybrid approach is proposed to investigate the sentiments of the public towards COVID-19 safety restrictions. Extensive experiments are performed to analyze the performance of machine learning using TF-IDF and BoW and deep learning models in comparison to the proposed EB-DT model. Experimental results show that the overall performance of machine learning classifiers is better with BoW features. The proposed EB-DT outperforms both machine learning and deep learning models with a 0.99 accuracy score when used with TF-IDF features. Moreover, this study uses topic modeling and 5 topics each for positive and negative tweets are extracted. Regarding the negative sentiments, with the mouth and nose covered by the facemask, people feel difficulty in breathing and condemn the restriction of wearing the facemask. On the other hand, positive tweets commend the use of facemask to be safe from viruses, and filters and provide clean air to breathe. Analyzing the change in tweets sentiments over time reveals that peoples' sentiments regarding COVID-19 restrictions have shifted from neutral to negative and positive. However, the tweets containing positive sentiments are substantially higher than tweets containing negative tweets.

**Concern 11:** Policy implications are weak.
**Response**: We would like to thank the reviewer for providing insightful comments. A separate Section, ‘Policy Implications’ has been added to the revised manuscript.

**Page 19 of the revised manuscript**

**Policy Implications**

This study is designed to analyze the reviews of the public regarding COVID-19 restrictions. Although such restrictions serve as the first line of defense and are initiated for the safety of the people from the pandemic, people do not share the same sentiment. Consequently, people show their willingness and dissent on social media platforms. Analyzing such views can provide useful insights into people's sentiments and provide an overall picture of what people think of these restrictions. Consequently, current policies about the COVID-19 restrictions can be drafted and revised accordingly. Additionally, the analysis of change in people’s sentiment over time can be beneficial to examine the impact of specific policies for a particular period. The study does not define any such policy or analyze it, it simply provides insight into people's sentiments. Since people's views and sentiments are deemed important, especially in developed countries, they do have an impact on the decision-making process and can be used to take corresponding actions.

**Concern 12:** Further research should be indicated.
**Response**: The reviewer's response is valuable and we added the future research directions in the revised manuscript.

**Page 20 of the revised manuscript**

A potential future work in this study is the utilization of part-of-speech tagging to remove the words which are not relevant to the sentiments such as nouns that do not contribute to the sentiment of the text. Another possible future work can be the identification of the fake accounts to reduce the bias in the dataset. The sentiment classification of social media texts enables governments to gain insights into the public opinion towards policy changes thus allowing them to make informed decisions. The results in this study are limited to the data related to Twitter users. Therefore, we plan on incorporating public opinions from a variety of social media platforms to provide generalizability to the proposed model.

**Concern 13:** Limitations of the study are not provided.
**Response**: Limitations of the study are added in the revised manuscript.

**Page 20 of the revised manuscript**

This study incorporates a dataset of a total of 8911 extracted tweets that may not be enough to analyze the performance of the deep learning algorithms as they tend to work well with large datasets. In addition, not all the extracted tweets are reliable as the number of fake accounts has drastically increased over time on social media platforms. This might introduce bias in the sentiments of the extracted tweets. Similarly, despite being the leading social media platform, Twitter does not contain all the views regarding COVID-19, and considering the views and comments from other social media platforms like Facebook, Reddit, etc. may change the results.

**Concern 14:** The English needs a proofreading by a native speaker.
**Response**: We would like to thank the reviewer for his valuable comments. English corrections and proofreading have been carried out as advised by the worth reviewer.

**Concern 15:** The editing does not follow the journal's guidelines.
**Response**: We update the manuscript according to the journal's guidelines.

**Concern 16:** The originality value of the study is limited.
**Response**: The reviewer's concern is valuable. We have emphasized the importance and originality of the study in the revised draft.

**Concern 17:** This is an applied exercise without a clear innovative intuition.
**Response**: Authors highly appreciate the reviewer's concern regarding intuition. In the Introduction, we have highlighted our intuition for analyzing people's sentiments regarding COVID-19 with its implications for policymaking. Contributions of the study have also been revised as follows

This study proposes a hybrid approach to perform sentiment analysis of tweets related to safety precautions for COVID-19 and makes the following contributions

- A unified framework of sentiment lexicon and machine learning classifier has been proposed where the proposed model is a voting classifier, extreme boosting-decision tree (EB-DT), and integrates extra tree classifier (ETC), gradient boosting machine (GBM), and DT models. For experiments, different sets of data are extracted from Twitter. Three different datasets are extracted, to be precise, for analyzing the change in peoples' sentiments over time.
- Machine learning models are also employed for performance comparison including AdaBoost classifier (AC), ETC, logistic regression (LR), random forest (RF), GBM, K nearest neighbors (KNN), support vector classifier (SVC), and DT which are used with term frequency-inverse document frequency (TF-IDF) and bag of words (BoW). Experiments also involve long short-term

memory (LSTM), convolutional neural network (CNN)-LSTM, and gated recurrent unit (GRU) with a large dataset collected from Twitter. The lexicon-based TextBlob is used to annotate the collected dataset.

- Topic modeling of positive and negative is carried out using the Latent Dirichlet allocation (LDA) algorithm to analyze the factors that negatively influence the safety precautions against COVID-19.
- The accuracy of the proposed framework regarding the sentiment classification is compared to a professional sentiment analysis app called 'sentiment viz app'.
